# Cytology of progressive multifocal leukoencephalopathy revisited: A case report with special reference to JC polyomavirus‐infected oligodendrocytes and astrocytes

**DOI:** 10.1111/cyt.13042

**Published:** 2021-08-14

**Authors:** Mieko Doi, Keisuke Ishizawa, Kei Ikeda, Kazuo Nakamichi, Yoshihiko Nakazato, Toshimasa Yamamoto, Atsushi Sasaki

**Affiliations:** ^1^ Division of Diagnostic Pathology Saitama Medical University Hospital Saitama Japan; ^2^ Department of Neurology Saitama Medical University Saitama Japan; ^3^ Department of Virology 1 National Institute of Infectious Diseases Shinjuku‐ku Japan

## Abstract

This article reports a case of progressive multifocal leukoencephalopathy in an immunocompromised female patient. The usefulness of cytology, as well as histology, is described, with special reference to JC polyomavirus‐infected oligodendrocytes and JC polyomavirus‐infected astrocytes.

## 1 INTRODUCTION

Progressive multifocal leukoencephalopathy (PML) is a central nervous system demyelinating disease which primarily affects immunocompromised individuals, including those with human immunodeficiency virus (HIV) infection, rheumatological diseases, and hematological malignancies.^1^, ^2^ The disease is caused by JC polyomavirus (JCV), and oligodendroglial intranuclear inclusions – which are identified by the presence of enlarged, hyperchromatic, “ground‐glass” nuclei – are the pathological hallmark of the disease.^1^, ^3^, ^4^, ^5^, ^6^, ^7^, ^8^ Recently, due to the advent of immunosuppressants and molecularly targeted drugs, it is not uncommon to encounter PML in routine diagnostic practice.^2^

In addition to histology, cytology was also of great help in reaching the diagnosis of PML in this patient. The cytological profiles are described here, with special reference to JCV‐infected oligodendrocytes and astrocytes, and the role of cytology in the diagnosis of PML is discussed.

## 2 CASE PRESENTATION

The patient was a female in her seventies. She was diagnosed as having autoimmune hemolytic anemia, and oral administration of prednisolone was initiated. She had been taking it for about 1 year, when, at the dose of 9 mg day^–1^, she noticed muscle weakness in her left upper limb. This symptom became worse, and she was admitted to our institution. On neurological examination, she showed left hemiparesis (4/5 on manual muscle testing). Deep tendon reflexes were not exaggerated, and pathological reflexes were not present. A series of blood investigations, including blood cell count, blood chemistry, and serum autoantibodies, were unremarkable, and the serum anti‐HIV antibodies were negative. Brain magnetic resonance imaging (MRI) disclosed irregularly shaped, patchy areas confined to the deep white matter of the right frontoparietal lobe without mass effect, which showed high intensity on T2‐weighted and fluid attenuated inversion recovery images and low intensity on T1‐weighted images. These areas showed high intensity on both diffusion weighted images and an apparent diffusion coefficient map. Gadolinium (Gd)‐enhancement was negative. These MRI findings suggested that leukoencephalopathy was the most likely diagnosis, although differentiation from infiltrating gliomas and multiple sclerosis (MS) was required. A stereotactic brain biopsy targeting the right parietal white matter was performed.

The intra‐operative touch imprint and squash smear materials were stained with hematoxylin & eosin (H&E) and Papanicolaou (Pap) for cytological evaluation. The remaining materials were embedded in paraffin, and the sections were stained with H&E and Klüver‐Barrera (KB), and immunostained for neurofilament protein (NFP) (mouse monoclonal, 2F11; 1:100; Dako), Iba‐1 (rabbit polyclonal; 1:1000; FUJIFILM Wako Pure Chemical Corporation), p53 (mouse monoclonal, DO‐7; 1:100; Dako), and SV40^1^, ^7^ (mouse monoclonal, PAb416; 1:100; Calbiochem) using heat‐treated antigen retrieval pretreatment. The immunostained sections were visualised with diaminobenzidine.

**FIGURE 1:**
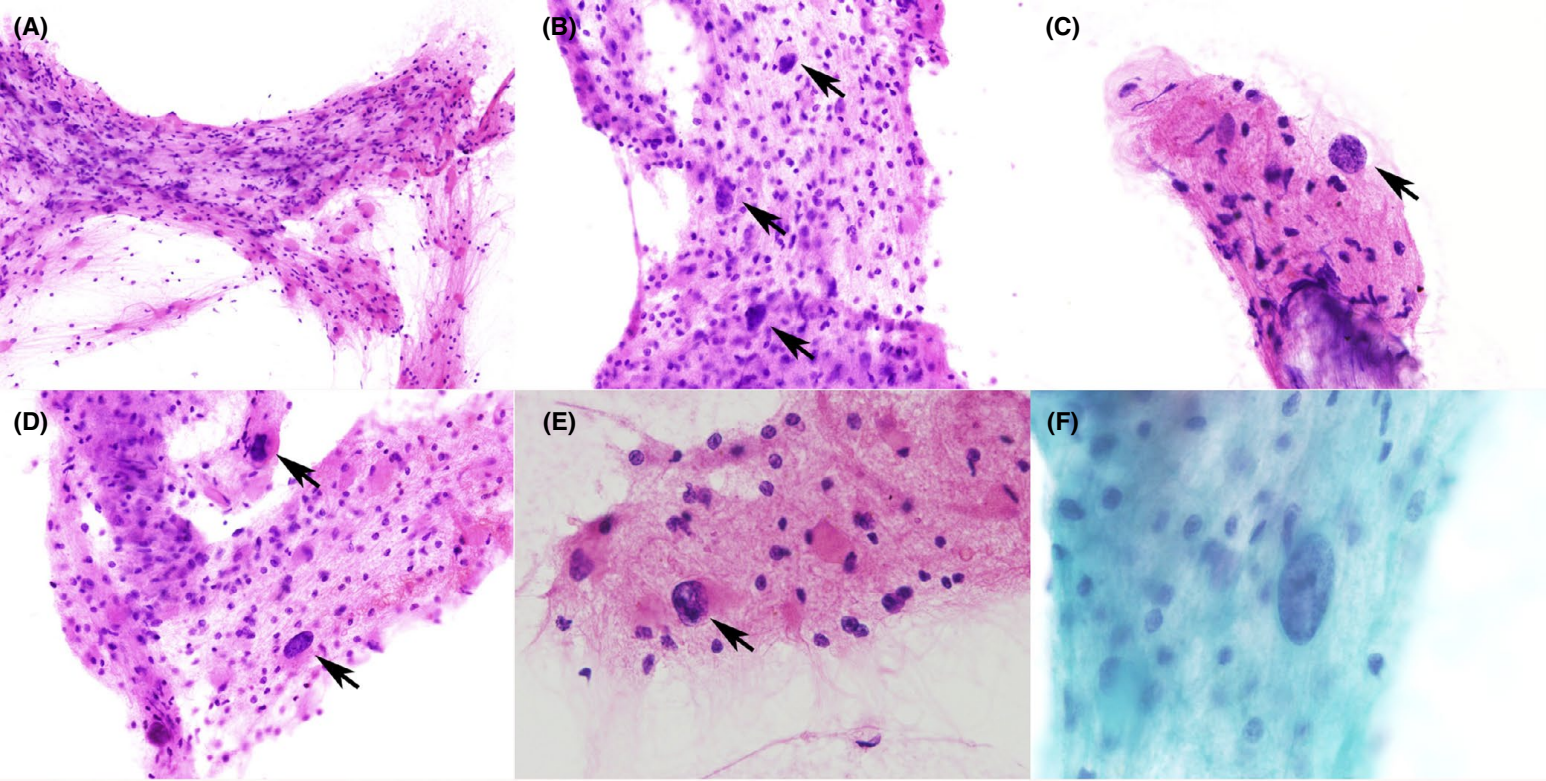
Cytological findings on squash smear specimens. (A) A cellular aggregate composed of variable cells (hematoxylin & eosin (H&E)). (B, C) Cells with enlarged, round/oval, and ground‐glass nuclei, appearing almost naked with scant cytoplasm. (arrows, H&E). (D, E) Cells with peripherally located, irregular nuclei and relatively plump, eosinophilic cytoplasm. (arrows, H&E). (F) An enlarged, ground‐glass nucleus (in the center; Papanicolaou). Magnification: (A) ×100, (B, D) ×200, (C, E) ×400, (F) ×600

On squash smear specimens, many cells were sampled. On H&E, many cellular aggregates composed of variable cells were noted (Figure 1A). In these, admixed with macrophages and reactive/gemistocytic astrocytes, another type of cell with enlarged, hyperchromatic, and ground‐glass nuclei was observed. Most of these cells harbored round/oval, almost naked nuclei and scant cytoplasm (Figure 1B,C), morphologically suggesting oligodendrocytes; however, a fraction of them harbored irregular, peripherally located nuclei and plump, eosinophilic cytoplasm, morphologically suggesting astrocytes (Figure 1D,E). The precise identity of large and multinucleated astrocytic cells, which were thus considered to be “Creutzfeldt cells”,^8^, ^9^ was not confirmed. Many cellular aggregates were also noted on Pap. Although the nuclei and cytoplasm were less clearly visible than on H&E, many cells with enlarged, hyperchromatic, and ground‐glass nuclei, which were reminiscent of those on H&E, were noted (Figure 1F). On touch imprint specimens only a few cells were present, and these specimens were therefore of little use for cytological evaluation.

**FIGURE 2:**
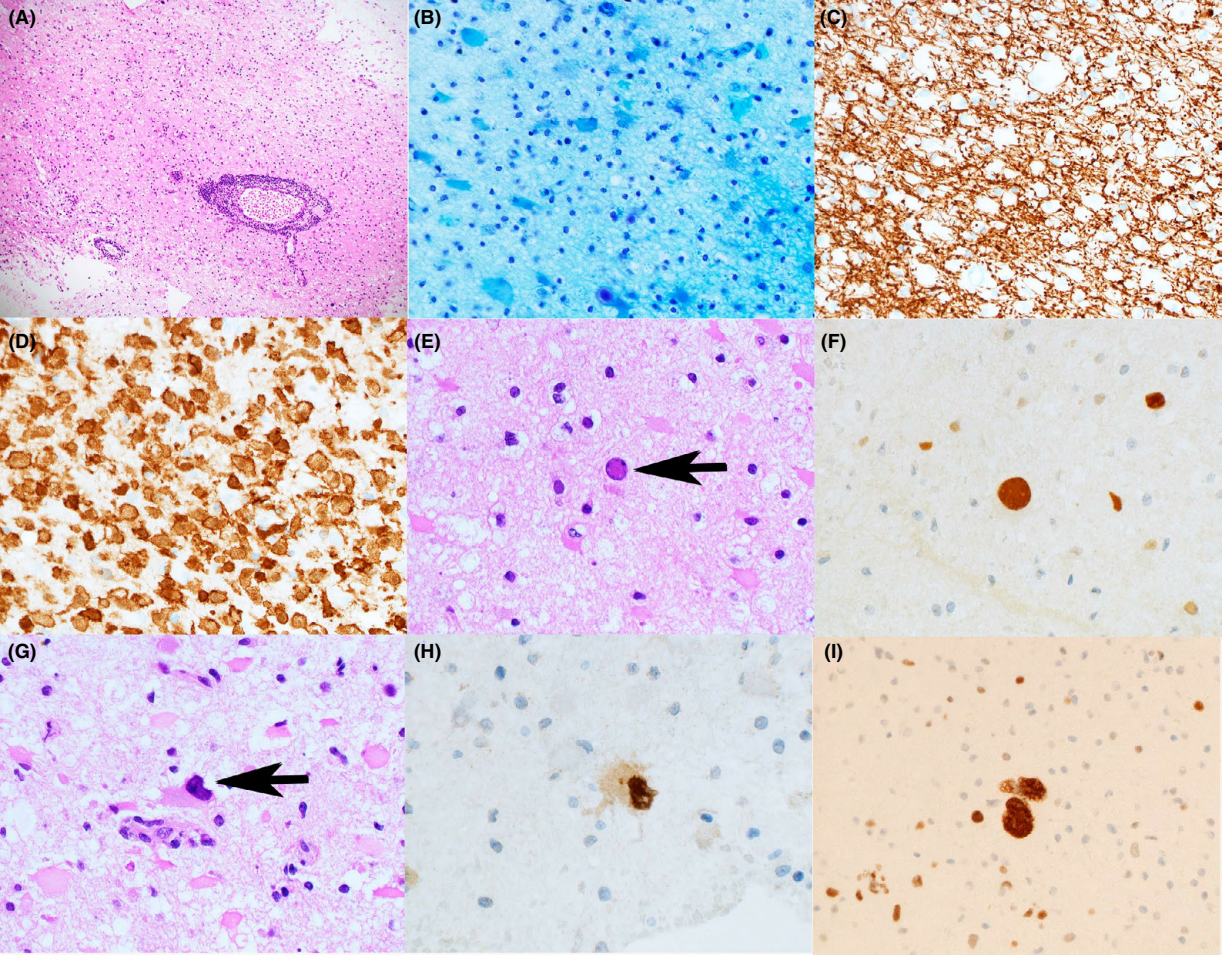
Histological findings. (A) Lesion showing marked infiltration of macrophages and astrocytes, and perivascular lymphoid cuffing (hematoxylin & eosin (H&E)). (B) Loss of myelin is evident. Many astrocytes that are non‐specifically stained blue are present. (Klüver‐Barrera); (C) Well‐preserved axons. Many vacuoles are visible, corresponding to infiltrating macrophages. (neurofilament protein (2F11) immunostain). (D) The infiltration of many macrophages is very clearly visualised via Iba‐1 immunostaining. (E) An enlarged, round, and ground‐glass nucleus without conspicuous cytoplasm. (arrow, H&E). (F) A nucleus that is immunoreactive for SV40 (in the center) and is morphologically similar to that shown in (E). Note SV40‐immunoreactive nuclei that are smaller (SV40 immunostain). (G) A cell with an irregular nucleus and plump, eosinophilic cytoplasm, exhibiting, upon close inspection, a ground‐glass nucleus. (arrow, H&E). (H) A cell which corresponds morphologically to that shown in (G) (in the center), whose nucleus is immunoreactive for SV40 (SV40 immunostain). (I) A number of cells, both enlarged and small, are immunoreactive for p53 (p53 (DO‐7) immunostain). Magnification: (A) ×100, (B‐D) ×400, (E‐I) ×600

On histology, the lesion showed marked infiltration of macrophages and astrocytes, and perivascular lymphoid cuffing (Figure 2A). A severe loss of myelin was observed (Figure 2B), while axons were well preserved (Figure 2C, NFP (2F11) immunostain), suggesting a demyelinating disease. On Iba‐1‐immunostained sections, the infiltration of macrophages was remarkable (Figure 2D). A closer look at the specimens disclosed enlarged, hyperchromatic, and ground‐glass nuclei that were similar to those observed on cytology (Figure 2E,G). Most of these nuclei were round/oval and almost naked, with scant cytoplasm (Figure 2E), and likely correspond to the cells pictured in Figure 1B,C. Some of these cells were immunoreactive for SV40 (Figure 2F, SV40 (PAb416) immunostain); however, others were observed that were morphologically irregular and peripherally located, with plump, eosinophilic cytoplasm (Figure 2G), likely corresponding to the cells pictured in Figure 1D,E. A number of these cells were also immunoreactive for SV40 (Figure 2H, SV40 (PAb416) immunostain). Interestingly, not only the enlarged nuclei but also the small ones were immunoreactive for SV40 (Figure 2F), as has previously been reported elsewhere.^5^ On p53‐immunostained sections, a number of p53‐immunoreactive nuclei, which were either enlarged or small, were noted (Figure 2I, p53 (DO‐7) immunostain). In general, the morphological details of nuclei were less clearly discernible on histology than on cytology.

The lumbar puncture, which had been performed some time before the brain biopsy, was found to be positive for variant JCV (3959 copies ml^–1^, prototype) in the cerebrospinal fluid (CSF) of the patient. After the introduction of combined mefloquine and mirtazapine therapy, the progression of the left hemiparesis stopped. About 1 year after admission, the titer of variant JCV in the CSF decreased to 978 copies ml^–1^, and the patient remained symptomatically unchanged.

## 3 DISCUSSION

Thus far, there have been several reports on the cytology of PML. A paper by Yu et al.^5^ reported the cytomorphology of PML in a series of 16 patients infected with HIV. This paper analyzed cytological parameters such as JCV‐infected oligodendrocytes, nuclear atypia of reactive astrocytes, and others (cellularity, etc). The JCV‐infected oligodendrocytes were characterised by enlarged nuclei with homogenous, smudged chromatin, imparting a “glassy” appearance, and little or no cytoplasm. By contrast, the cells with eccentrically placed nuclei, abundant fibrillary cytoplasm, and mild to moderate nuclear atypia, but without observations of chromatin smudging or intranuclear inclusions, were classified as reactive astrocytes. However, in a previous paper detailing the cytology from two cases of PML,^3^ a population of atypical cells with abundant dense eosinophilic cytoplasm forming multiple cytoplasmic processes was described; the nuclei of these were enlarged, hyperchromatic, multinuclear, and smudged, lacking discrete chromatin structures. This population of atypical cells could be JCV‐infected astrocytes, but the authors did not mention whether or not they were. In our investigations of the literature relating to atypical astrocytes, those that have been documented on cytology of PML have been placed in the category of “reactive astrocytes.”^5^, ^6^, ^10^ However, as was seen in the present case, JCV‐infected astrocytes, whose nuclei are reminiscent of those of JCV‐infected oligodendrocytes, do exist in PML. It has been previously reported elsewhere that astrocytes can be infected with JCV.^11^, ^12^ Although JCV‐infected astrocytes may account for a small proportion of JCV‐infected cells in PML, relative to JCV‐infected oligodendrocytes, the recognition of JCV‐infected astrocytes likely enhances the diagnostic accuracy of PML. Moreover, although both JCV‐infected astrocytes and JCV‐infected oligodendrocytes are recognisable on cytology and histology alike, the cytology could provide better samples for the recognition of the characteristic nuclei.

The recognition of JCV‐infected astrocytes is also important for differentiating PML from infiltrating astrocytomas. In the present study, many atypical cells, some of which were conspicuously astrocytic, were noted on both cytology and histology, potentially masquerading as infiltrating astrocytomas. This feature was all the more confusing due to the presence of p53‐immunoreactive nuclei on immunohistochemistry (Figure 2I), because p53‐immunoreactive nuclei are inseparably associated with infiltrating astrocytomas.^13^ However, on cytology, as was noted in the present study, possible JCV‐infected astrocytes harboring ground‐glass nuclei can be observed in PML, and this feature, along with JCV‐infected oligodendrocytes harboring ground‐glass nuclei as well as marked infiltration of macrophages,^1^, ^5^, ^6^, ^7^ can lead to a possible/probable intraoperative diagnosis of PML. On the other hand, nuclear pleomorphism, mitotic figures, glomeruloid vascular proliferation (endothelial proliferation), necrotic debris, and a relative paucity of macrophages compared to PML, are cytological features that favour the intraoperative diagnosis of infiltrating astrocytomas.^6^, ^9^ In an immnohistochemical context, it should be borne in mind that PML, despite its non‐neoplastic nature, is associated with nuclear p53‐immunoreactivity^7^, ^14^, as are infiltrating astrocytomas.^13^ This knowledge of p53‐immunoreactivity on immunohistochemistry in PML could prevent the misdiagnosis of infiltrating astrocytomas and strengthen the diagnosis of PML. Recently, the identification of a number of immunohistochemical markers, including not only p53 but also mutated isocitrate dehydrogenase 1 (IDH1R132H) and alpha‐thalassemia X‐linked intellectual disability (ATRX), and molecular markers including epidermal growth factor receptor (EGFR) using, for example, fluorescence in situ hybridisation, has been shown to be an indispensable tool for the diagnosis of infiltrating astrocytomas^13^; this immunohistochemical and molecular testing will be of great help in the differential diagnosis of PML vs infiltrating astrocytomas.

The differential diagnosis of PML vs other demyelinating diseases, most notably MS, is also worthy of discussion here. Clinically, MS usually presents in young (up to middle‐aged,) immunocompetent persons, about two thirds of whom are women.^15^ The variable neurological symptoms and signs of MS are typically disseminated in time and space.^15^ Neuroradiologically, the MS lesions, which are ovoid‐shaped and spatially multiple, are often located periventricularly or, to a lesser extent, subcortically,^8^, ^15^ and the “open ring sign” on Gd‐enhanced MRI is considered to be fairly specific to demyelinating diseases, including MS.^16^ These clinical and neuroradiological features of MS are considerably different from those of PML. Moreover, in a cytological context, many macrophages and reactive astrocytes, the latter of which are sometimes large and multinucleated (“Creutzfeldt cells”), are noted in PML and MS alike,^8^, ^9^ potentially misleading the differential diagnosis of PML vs MS; however, the ground‐glass nuclei seen on cytology in PML are altogether absent in MS. These cytological features, along with the differing clinical and neuroradiological features, are helpful for the differential diagnosis of PML vs MS. It should be borne in mind, however, that because of the introduction of molecularly targeted drugs for the treatment of MS, the number of cases of PML overlying preexistent MS is on the rise.^2^

The clinical presentation in immunocompromised individuals,^1^, ^2^ white matter lesions with little or no enhancement/mass effect on neuroimaging studies,^7^, ^8^, ^17^ detection of variant JCV in the CSF, cytological and histological recognition of ground‐glass nuclei in both JCV‐infected oligodendrocytes and JCV‐infected astrocytes, and immunohistochemical detection, in particular, of SV40‐immunoreactivity or even p53‐immunoreactivity, strongly suggest the diagnosis of PML. The present study, in conjunction with previous reports,^3^, ^4^, ^5^, ^6^ confirms the usefulness of cytology for the diagnosis of PML. It also suggests the importance of cytological and histological recognition not only of JCV‐infected oligodendrocytes but also of JCV‐infected astrocytes in the diagnosis of PML.

## CONFLICT OF INTEREST

The authors have no conflict of interest to declare.

## AUTHOR CONTRIBUTIONS

M.D., K.I. (the second author), and A.S. evaluated the cytological and histological profiles of the specimens, made the pathological diagnosis of PML, and drafted the manuscript. K.I. (the third author), Y.N., and T.Y. took care of the patient in the clinical setting and made a great contribution to the diagnosis and treatment of the patient. K.N. analyzed the cerebrospinal fluid of the patient and disclosed the presence of variant JCV, contributing greatly to the definite diagnosis of PML.

## CONSENT

Brain biopsy was performed as a routine diagnostic test with the written informed consent of the patient. This study was conducted according to the regulations of the Institutional Review Board (IRB) of Saitama Medical University Hospital.

## Data Availability

No additional data are available since this is a case report of a single case.
